# Sensitivity and specificity of diagnostic tests for Lassa fever: a systematic review

**DOI:** 10.1186/s12879-019-4242-6

**Published:** 2019-07-19

**Authors:** Noah Fongwen Takah, Polina Brangel, Priyanka Shrestha, Rosanna Peeling

**Affiliations:** 1 International Diagnostics Centre Africa, Addis Ababa, Ethiopia; 2 London Centre for Nanotechnology, University College London, London, UK; 3 International Diagnostics Centre, Clinical Research Department, London School of Hygiene and Tropical Medicine, Keppel Street, London, WC1E 7HT UK

**Keywords:** Lassa, Diagnostics, Sensitivity, Specificity

## Abstract

**Background:**

Lassa fever virus has been enlisted as a priority pathogen of epidemic potential by the World Health organization Research and Development (WHO R & D) Blueprint. Diagnostics play a crucial role in epidemic preparedness. This systematic review was conducted to determine the sensitivity and specificity of Lassa fever diagnostic tests for humans.

**Methods:**

We searched OVID Medline, OVID Embase, Scopus and Web of Science for laboratory based and field studies that reported the performance of diagnostic tests for Lassa fever in humans from 1 January 1990 to 25 January 2019. Two reviewers independently screened all the studies and included only studies that involved the evaluation of a Lassa fever diagnostic test and provided data on the sensitivity and specificity. The quality of the studies was assessed using the QUADAS-2 criteria. Data on the study location, study design, type of sample, index test, reference tests and diagnostic performance were extracted from the studies.

**Results:**

Out of a total of 1947 records identified, 1245 non-duplicate citations were obtained. Twenty-six (26) full-text articles examined which identified 08 studies meeting pre-defined criteria. Only one study was a field evaluation study. The sensitivity and specificity of the point of care (RDT) against the Nikisins qPCR were 91.2%(95% CI:75.2–97.7) and 86%(95% CI: 71.4–94.2) at temperatures 18–30 °C, while the sensitivity and specificity of the single IgM ELISA assay against standard RT-PCR were 31.1%(95%CI: 25.6–37) and 95.7%(95%CI:92.8–97.7). The sensitivity of the combined ELISA Antigen/IgM assay(against virus isolation), the recombinant IgM/IgG ELISA(against standard RT-PCR), and the IgM/IgG immunoblot(against IFA) were 88%(95%CI:77–95), 25.9%(95%CI:20.8–31.6), and 90.7%(95%CI:84.13–97.27) respectively. The specificity of the combined ELISA Antigen/IgM assay(against virus isolation), the recombinant IgM/IgG ELISA(against standard RT-PCR), and the IgM/IgG immunoblot(against IFA) were 90%(95%CI:88–91), 100%(95%CI:98.2–100), and 96.3%(95%CI:92.2–100) respectively.

**Conclusion:**

Lassa fever has assays for antigenaemia, IgM, IgG and PCR detection. The RDT reportedly performed well but more data are needed from other countries and at temperatures above 30 °C. Most combined immunoassays perform better than the single IgM. Multiplex and pan-Lassa assays are needed. More well conducted field studies are needed.

**Trial registration:**

Prospero registration number: CRD42018091585.

**Electronic supplementary material:**

The online version of this article (10.1186/s12879-019-4242-6) contains supplementary material, which is available to authorized users.

## Background

Lassa fever is an acute and potentially fatal hemorrhagic illness caused by the Lassa Fever Virus [1]. The disease has been shown to be endemic in several West African countries including Benin, Guinea, Liberia, Côte d’Ivoire, Mali, Nigeria, and Sierra Leone [2]. The disease is also important in global health security as reflected in the inclusion of the Lassa fever virus in the World Health Organization Research and Development (WHO R&D) Blueprint as a priority pathogen of epidemic potential [3]. Studies have reported the number of clinical infections to be approximately 100,000–300,000 in West Africa per year, with approximately 5000 deaths [4, 5]. The recent outbreak of the Lassa epidemic in Nigeria resulted in 327 cases of Lassa fever (324 confirmed and three probable cases) with 72 deaths (case fatality ratio of 22%) from 1 January to 10 February 2019. The WHO estimates a Lassa case fatality rate of 1, and 15% amongst patients hospitalized with severe illness [6].

Lassa fever is a zoonotic infection that can be transmitted to humans through contact with virus-infected rodent excreta via eating rodent-contaminated food, exposure to contaminated objects, and inhalation of tiny particles in the air contaminated with virus-infected rodent excretions [7]. Though not widely observed in epidemic outbreaks, human-to-human transmission can also occur through contact with fluids of infected persons in health care settings through poor infection control measures [8]. Diagnostics play a pivotal role in the control of an outbreak of Lassa fever by: permitting early diagnosis which can necessitate prompt antiviral therapy and reduce morbidity and mortality; assisting in the tracking of community contacts as well as providing the true picture of the epidemic [9–11].

Despite the relevance of diagnostics in the response to Lassa fever outbreaks, the availability of Lassa fever diagnostics is limited for several reasons. Clinically, most Lassa fever patients are asymptomatic, and even when symptoms are present, they can be non-specific [12]. There are also enormous challenges faced in developing an efficacious assay due to the complexity of the Lassa virus sequence diversity [13]. Furthermore, the collection, storage and handling of specimens for Lassa fever requires biosafety level precautions similar to the Ebola virus (Ref). The need for high containment safety requirements and the scarcity of high containment laboratories in many parts of the World may have led to limited Lassa fever assay development and validation studies [14]. However, with the World Health Organization (WHO) call for early diagnostic tests for Lassa [15] and the inclusion of Lassa fever virus as a priority pathogen of epidemic potential in the WHO Research and Development (WHO R &D) [3, 5], it is necessary to systematically review all the diagnostic tests available, so that gaps in diagnostic research and development can be identified that can guide innovation in R & D.

Raabe and Koehler conducted a minireview that provides an overview of the currently available diagnostic tests for Lassa [14]. Given that it was minireview, it was clear that the search was not systematic with no quality assessment of the included studies. In addition, the review doesn’t provide complete data on the sensitivity and specificity of the diagnostic tests used. Based on the scarcity of systematic reviews with regards to Lassa diagnostics, we conducted this systematic review to determine the sensitivity and specificity of tests available for the diagnosis of Lassa fever in humans. This study will be important in identifying the weaknesses, strengths and applications of the diagnostic assays available for Lassa fever.

## Methods

### Search strategy and identification of studies

This was a systematic review of the diagnostic accuracy of Lassa fever tests used for humans. The review was registered in PROSPERO (Registration number: CRD42018091585) [16] and reported in accordance with the Preferred Reporting Items for Systematic Reviews and Meta-analyses (PRISMA) check list [17]. Literature search strategies were developed by two reviewers (NFT and PB) with the help of a librarian at the London School of Hygiene and Tropical Medicine with expertise in systematic review searching. The search strategies are shown in Additional file 1: Table S1 and Additional file 2: Table S2. Two reviewers (NFT and PB) independently searched MEDLINE, EMBASE, the Cochrane Central, Cochrane database for systematic reviews, Scopus, Web of Science, Google Scholar. Additional pertinent citations were identified through bibliographies of retrieved studies. Abstracts were imported into Mendeley and independently screened by two reviewers NFT and PB according to standard inclusion and exclusion criteria. All studies identified for full manuscript review were assessed independently by two reviewers (NFT and PB) against inclusion criteria. Any discrepancies were settled by a round table discussion and consultation with the third reviewer (RP).

### Selection criteria

We included case-control, cross-sectional, cohort studies published between 1 January 1990 and 25 January 2019, with primary purpose of evaluating Lassa fever test accuracy using any clinical specimen type. We also included studies reporting original data from patient specimens in all age groups, studies that reported the laboratory, clinical/field evaluation of a diagnostic tests in humans; studies that reported the sensitivity and specificity with a reference standard. We excluded: articles in languages other than English; conference abstracts, comments or review papers; studies only reporting sensitivity or specificity without reference standards; studies using commercially prepared reference panels. We excluded studies not involving human subjects.

### Data extraction and quality assessment

Two reviewers (NFT and PS) independently extracted data and reached agreement on the following variables in the data extraction sheet: study author and year; study location and design; patient age range; name and format of the test; reference test; type and number of specimens tested; type of evaluation (clinical/field vs laboratory); sensitivity and specificity with 95% confidence intervals; phase of infection during which sample were collected; funding source and any reported conflict of interest. We calculated the 95% confidence intervals for the studies reporting only point estimates.

Study quality was evaluated using the Quality Assessment of Diagnostic Accuracy Studies (QUADAS)-2 tool [18], which evaluates risk of bias (patient selection, index test, reference standard, and patient flow through) and applicability concerns.

### Data synthesis and analysis

We could not conduct a meta-analysis because: the number of studies for each diagnostic test type was not enough to pool together meaningful data; and the authors were not consistent in the reference standards used in the evaluation of the index tests. Therefore, we carried out only a qualitative synthesis of the studies, taking into consideration the weakness and strengths in the methodologies of evaluation of the diagnostic technologies, that may have influenced the sensitivity and specificity results obtained.

## Results

### Study selection and characteristics

**Fig. 1:**
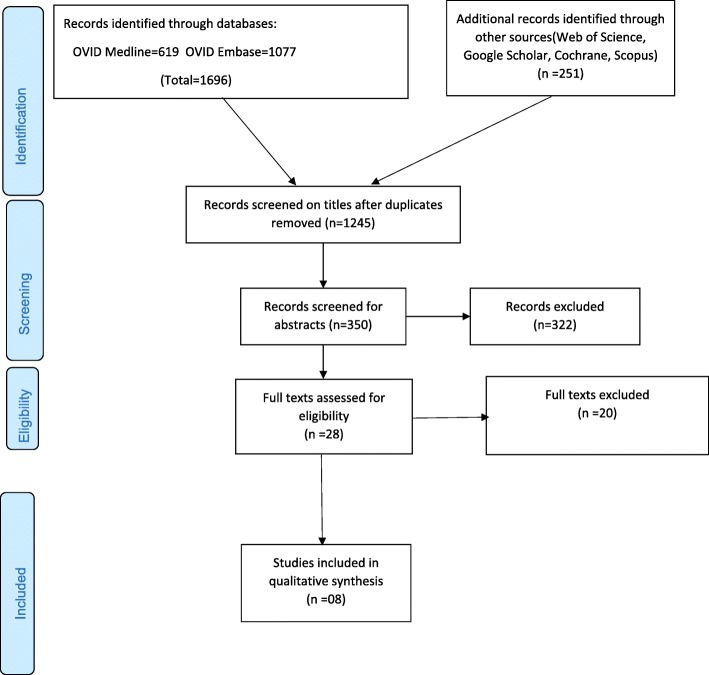
PRISMA flow diagram showing databases searched, screening and inclusion of studies

A total of 1245 non-duplicate citations were identified, and 26 full-text articles examined which identified 08 studies meeting pre-defined criteria (Fig. 1). Of the included studies, 2 were conducted with patients from Sierra Leone only, 2 were conducted with patients from Nigeria only, while in 4 studies, patients were enrolled from multiple countries (Nigeria, Sierra Leone and Guinea) and in two studies, samples from patients in Germany were used (Table 1). In most of the studies (05), samples were collected from patients suspected of having Lassa fever. However, one study enrolled the close contacts of patients suspected of having Lassa fever, one study enrolled all patients with fever of unknown origin and hemorrhage, one study enrolled healthy adult blood donors and one study enrolled only Lassa confirmed cases (Table 1).

**Table 1:** Characteristics of included studies: study population and design

Author and reference	Year Published	Journal	Study Country	Study population	Study design	Patient Age Range
Boisen et al. [19]	2018	Scientific Reports	Sierra Leone	Suspected Lassa fever patients and their contacts	Prospective cohort	not stated
Boisen et al. [19]	2018	Scientific Reports	Sierra Leone	Suspected Lassa fever patients and their contacts	Prospective cohort	not stated
Bausch et al. [20]	2000	Journal of Clinical Virology	Sierra Leone and Guinea	Suspected Lassa fever patients	Prospective cohort	not stated
Gabriel et al. [21]	2017	PLOS Neglected Tropical Diseases	Nigeria, Ghana and Germany	Suspected Lassa fever patients	Prospective cohort	not stated
Ibekwe et al. [22]	2012	Nigerian Medical Journal	Nigeria	Clinically diagnosed patients and referred suspected patients	Case-control	11–61 years
Meulen et al. [23]	1998	Journal of Clinical Microbiology	Guinea and Liberia and Germany (Hamburg)	Patients with fever of unknown origin and hemorrhage	Cross-sectional	not stated
Emmerich et al. [24]	2006	Journal of Clinical Virology	Nigeria, Liberia, Sierra Leone and Guinea	Healthy adult blood donors	Cross-sectional	18–53
Olschlager et al. [25]	2010	Journal of Clinical Microbiology	Liberia and Nigeria	Laboratory confirmed Lassa patients	Cross-sectional	not stated
Trappier et al. [26]	1993	American Journal of Tropical Medicine and Hygiene	Sierra Leone	Suspected Lassa fever patients	Cross-sectional	not stated

Only two studies specified the age range of the patients from whom samples were taken. Studies were prospective, cross-sectional or case-control, and predominantly in the laboratory setting (Table 1). Only 01 study compared a rapid diagnostic test (RDT) against an immunoassay and a molecular reference standard (Table 2). This study was conducted in Sierra Leone and involved the field of evaluation of the Recombinant Lassa Virus RDT. Five studies compared immunoassays against virus isolation or molecular reference standards, while two studies compared molecular assays to virus isolation as reference standard (Table 2).

**Table 2:** Characteristics of studies: test name, reference test, type of evaluation and diagnostic performance

Author	Test Name	Reference Test	Type of Samples	Number of Samples Tested	Type of evaluation	Sensitivity(95% CI)	Specificity(95% CI)
Boisen et al	Recombinant Lassa Virus Rapid Diagnostic Test (ReLASV RDT)	Nikisins qPCR	Serum and plasma	451	Field evaluation	91.2(75.2–97.7)	86(71.4–94.2)
		ReLASV Ag ELISA	Serum and plasma	451	Field evaluation	94.8(81.3–99.1)	100(88.6–100)
Boisen et al	Recombinant Lassa Virus Antigen ELISA(ReLASV Ag ELISA)	Nikisins qPCR	Serum and plasma	451	Field evaluation	91.2(75.2–97.7)	86(71.4–94.2)
Bausch et al	Combined ELISA Antigen/IgM assay	Virus Isolation	Serum	305	Laboratory	88(77–95)	90(88–91)
	Indirect Immunofluorescent antibody test	Virus Isolation	Serum	305	Laboratory validation	70(57–81)	88(85–90)
Gabriel et al	IgM ELISA	Standard RT-PCR	Serum	270	Laboratory	31.1(25.6–37)	95.7(92.8–97.7)
	Recombinant IgM/IgG ELISA	Standard RT-PCR	Serum	270	Laboratory	25.9(20.8–31.6)	100(98.2–100)
Ibekwe et al	IgM ELISA	Standard RT-PCR	Serum	72	Laboratory	57(41.05–72.95)	77(63.06–90.94)
Meulen et al.	IgM/IgG Immunoblot	Indirect Immunofluorescent assay(IFA)	Serum	157	Laboratory	90.7(84.13–97.27)	96.3(92.2–100)
Emmerich et al	Reverse IgG ELISA	IFA	Serum	643	Laboratory	100(99–100)	99(98–100)
Olschlager et al	GPC RT-PCR(Qiagen)	Virus isolation	Serum and CSF	77 + (1CSF sample)	Laboratory	100(99–100)	
Trappier et al	Conventional PCR	Virus Isolation	Serum	195	Laboratory	66(55–77)	78(69.5–84.5)

Four (04) out of the six (06) studies that involved the evaluation of antigen and/or antibody assay clearly stated the phase of Lassa infection during which samples were collected from patients and tested. Most of the samples were tested in the acute phase. One study used convalescent samples (Table 3). Two studies did not state their funding source (Table 3). Table 4 shows the excluded full texts, the assays and the reasons for exclusion.

**Table 3:** Characteristics of included studies cont’d: phase of infection and funding source

Author Last	Test Name	Phase of infection	Funding source
Boisen et al	Recombinant Lassa Virus Rapid Diagnostic Test (ReLASV RDT)	Acute phase	NIH (National Institute for Health). Possible conflict of interest with Viral Hemorrrhagic fever Consortium.
Boisen et al	Recombinant Lassa Virus Antigen ELISA (ReLASV Ag ELISA)		NIH (National Institute for Health). Possible conflict of interest with Viral Hemorrrhagic fever Consortium.
Bausch et al	Combined ELISA Antigen/IgM assay	Acute phase. Convalescent samples collected but data on testing not given.	American Association of Schools of Public Health (AASPH) & Tulane University
	Indirect Immunofluorescent antibody test		
Gabriel et al	IgM ELISA	Not clear	European Fund for regional development
	IgM/IgG ELISA		
Ibekwe et al	IgM ELISA	Acute phase	No funding source
Meulen et al.	IgM/IgG Immunoblot	Not clear	Not stated
Emmerich et al	Reverse IgG ELISA	Convalescent samples (from survivors)	Bundesamt f¨ur Wehrtechnik und Beschaffung
Olschlager et al	GPC RT-PCR(Qiagen)	Not clear	Bundesamt f¨ur Wehrtechnik und Beschaffung
Trappier et al	Conventional PCR	Not clear	Not stated

**Table 4:** Excluded full texts and the reasons for exclusion

Study	Assay	Reason
1- Fukuma et al, 2011 [27]	Reverse Transcription LAMP	No data on sensitivity and specificity. Just talks about assay development
1- Fukushi et al, 2012 [28]	Serological assays	Review. No data on performance
3- Pang et al, 2014 [29]	Multiplex one step Real-Time Taqman qRT-PCR	No data on sensitivity and specificity given. Not a lab or field evaluation.
4- Das et al, 2015 [30]	Multiplex PCR/LDR assay	Not an evaluation. No data on sensitivity and specificity
5- Jones A et al, 2011 [31]	Handheld multiplex POC diagnostics	No data on sensitivity and specificity
6- Trombley et al, 2010 [32]	RT-TaqMan PCR	Not an evaluation study. No data on sensitivity and specificity
7- Drosten et al, 2002 [33]	SYBR-green real time RT-PCR	Not a lab or field evaluation.
8- Bukbuk et al, 2014 [34]	Recombinant NP(rNP) IgG ELISA	No data on sensitivity and specificity
9- Barber et al, 1990 [35]	Recombinant ELISA	Not evaluated in humans. No data on sensitivity and specificity
10- Li et al, 2009 [36]	Fluorescent microbeads based multiplex assay	No data on performance
11- Saijo et al, 2007 [37]	Ag-Capture ELISA	No data on the performance
12- Salvato et al, 2018 [38]		Review
13- Satterly et al, 2016 [39]	Ag and IgM Capture(MAGPIX)	
14- Olschlager et al, 2012 [40]	RT-PCR/hybridization assay	No data on sensitivity and specificity
15- Vieth et al, 2007 [41]	RT-PCR	Not an evaluation study
16- Leski et al, 2009 [42]	High Density Resequencing microarray	No data on sensitivity and specificity.
17- O’Hearn et al, 2016 [43]	IgG(MAGPIX)	No data on sensitivity and specificity
18- Demby et al, 1994 [44]	Standard RT-PCR	Not an evaluation study
19- Sebba D et al, 2018 [45]	Multiplex POC test for Lassa, Ebola and Malaria	None of the 276 clinical samples tested with the 3-plex assay were independently tested for LASV.
20- Koehler et al, 2018 [46]	Highly Multiplex Broad Pathogen detection assay	No data on sensitivity and specificity.

### Assessment of quality of studies

**Fig. 2:**
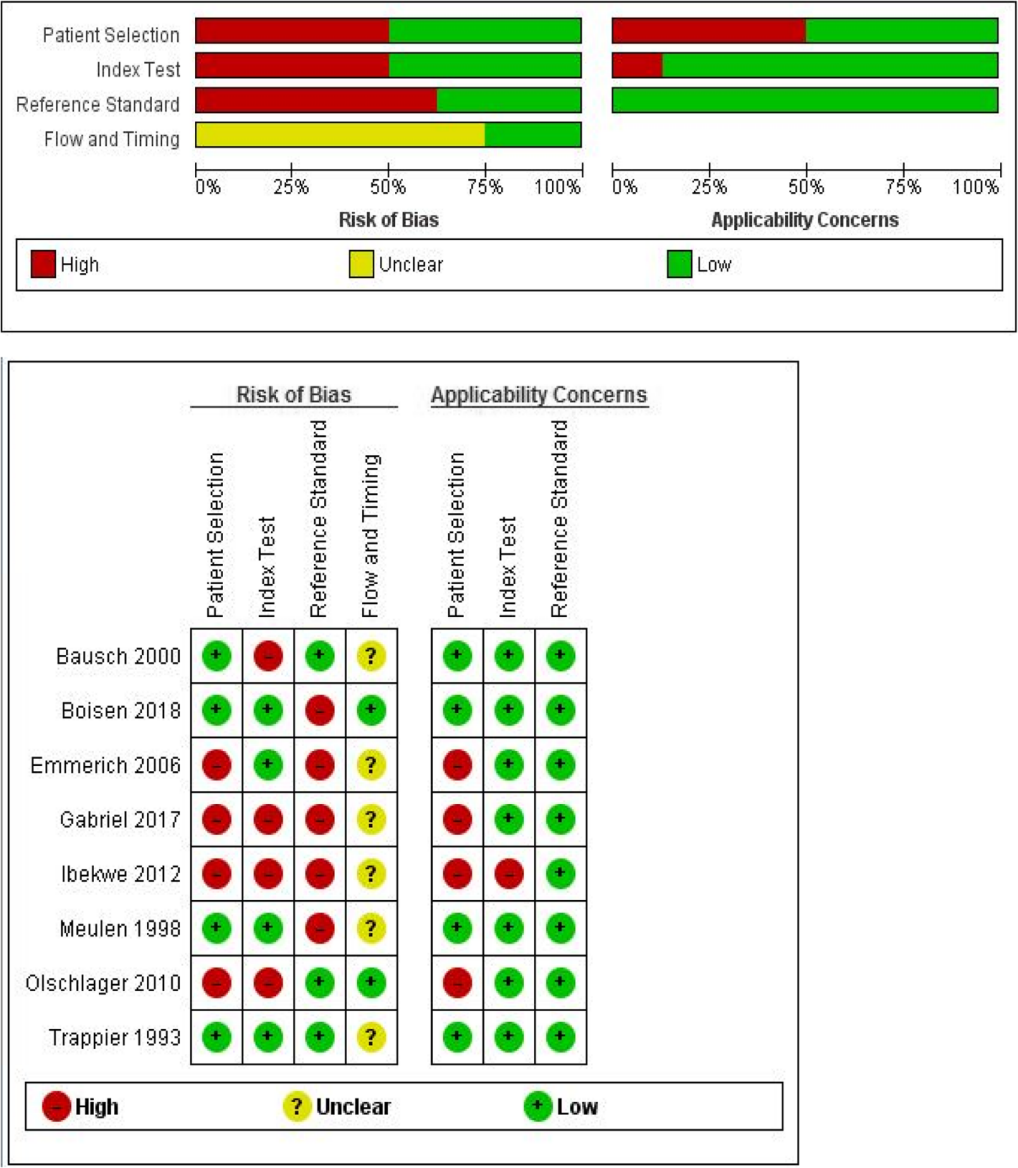
Results of the quality assessment of studies using the QUADAS-2 tool

The results of the QUADAS-2 assessment for risk of bias of each study, including summary figures, are shown (Fig. 2). Bias in patient selection was generally attributable to a case-control study design, or from enrolment of highly selected participants such as only patients with laboratory confirmed Lassa fever and healthy adult blood donors. Risk of bias from the index test was most commonly because they were conducted and interpreted with the knowledge of the results of the reference standard. There was an unclear risk of bias in flow and timing because majority (75%) of studies did not specify the exact time interval between performance of the index and reference assay. In up to 65% of the studies, there was high risk of bias in the reference standard because virus culture was not used. However, the applicability of the reference standard was of low concern because the target condition that was defined matched the review question.

### Diagnostic accuracy of tests for Lassa

#### For rapid diagnostic test (RDT)

Only one study assessed the accuracy of a rapid test for the diagnosis of Lassa fever [19]. This RDT evaluated is the dipstick recombinant Lassa virus (ReLASV) assay which is a lateral flow immunoassay based on paired monoclonal antibodies to the Josiah strain of the Lassa virus. This study was a prospective cohort study conducted in Sierra Leone and involved the use of 451 serum and plasma samples from patients suspected of having Lassa fever. The reference standards used were Recombinant Antigen ELISA (Enzyme Linked Immunosorbent Assay) and the Nikisins qPCR (quantitative polymerase chain reaction). Compared with the ReLASV antigen ELISA as the standard, the sensitivity and specificity of the test were 94.1%(95%CI:78.9–99.0%) and 83.7% (68.7–92.6%) respectively at temperatures 18–30 °C. Meanwhile, compared with the Nikisins qPCR, the sensitivity and specificity were 91.2%(95%CI: 75.2–97.7) and 86%(95%CI:71.4–94.2) respectively at temperatures 18–30 °C. Visual interpretation was possible within 15–25 min.

#### For immunoassays

Six studies evaluated the performance of immunoassays against different reference standards. Three of the studies assessed the performance of combined immunoassays [19–24]. The three combined immunoassays and their reference standards include: The combined ELISA Antigen/IgM assay against virus culture [20]; the combined IgM/IgG ELISA against standard RT-PCR [21]; the combined IgM/IgG Immunoblot against Indirect Immunofluorescent assay(IFA) [23].

The sensitivity of the combined ELISA Antigen/IgM assay, the IgM/IgG ELISA, and the IgM/IgG immunoblot were 88%((95%CI:77–95), 25.9%(95%CI:20.8–31.6), and 90.7%(95%CI:84.13–97.27) respectively. The specificity of the combined ELISA Antigen/IgM assay, the IgM/IgG ELISA, and the IgM/IgG immunoblot were 90%((95%CI:88–91), 100%(95%CI:98.2–100), and 96.3%(95%CI: 92.2–100) respectively. In total, the studies involving the evaluation of the combined immunoassays used 732 serum samples.

Three studies evaluated a single immunoassay against their reference standards as well. These single immunoassays include: The Recombinant Antigen ELISA assay against the Nikisins qPCR [19]; the IgM ELISA against RT-PCR [22]; and the Reverse IgG ELISA against IFA [24]. The sensitivity of the Recombinant Ag ELISA, IgM ELISA and Reverse IgG ELISA were 91.2%(95%CI:75.2–97.7), 57%(95%CI:11.05–72.95) and 100%(95%CI:99–100) respectively. The specificity of the Recombinant Ag ELISA, IgM ELISA and Reverse IgG ELISA were 86%(95%CI:71.4–94.2), 77%(95%CI:63.06–90.94) and 99%(95%CI:98–100) respectively. The studies that evaluated the single immunoassays used a total of 1166 serum samples.

Gabriel et al [21] also evaluated the single IgM ELISA assay alongside the evaluation of the combined IgM/IgG assay. The sensitivity and specificity of the single IgM assay against RT-PCR from this study were 31.1(95%CI: 25.6–37) and 95.7(95%CI: 92.8–97.7) respectively.

#### For molecular tests

Two studies reported the diagnostic performance of molecular assays [25, 26]. The molecular assays evaluated in the laboratory were the Glycoprotein Precursor (GPC) gene specific RT-PCR assay [25] and the conventional PCR [26]. The GPC RT-PCR/2007 assay was validated with 77 serum samples and 1 CSF sample from patients with laboratory- confirmed Lassa fever. Compared with virus culture, the sensitivity of the assay was 100%(95%CI:99–100). However, the sensitivity and specificity of the conventional PCR assay were 66%(95%CI: 55–77) and 78%(95%CI:69.5–84.5) respectively. No study evaluated the diagnostic performance of a multiplex assay for detection of many Lassa fever strains.

## Discussion

### Study findings

The findings from this first ever conducted systematic review on the diagnostic performance of Lassa fever assays show that of the studies included, the authors reported different assays available for the detection of the Lassa fever virus ranging from a point of care recombinant antigen test, combined and single ELISA immunoassays and molecular assays. The performance of combined immunoassays was higher than the single IgM ELISA assays, making the use of single immunoassays of IgM ELISA unsuitable for the screening of Lassa fever patients. Furthermore, we found no study that evaluated and reported the sensitivity and specificity of a multiplex assay for detection of the different strains of Lassa virus in West Africa.

### Reference standards for Lassa fever diagnosis

In our study, we found that different reference standards were used in the evaluation of diagnostic performance of Lassa tests. This variation in reference standards ranging from immunoassays to real time RT-PCR assays is a clear indication that there is still no uniformly accepted clinical reference standard besides virus culture. Even though the high sensitivity and specificity of real time RT-PCR assays make them suitable for this role [14], there is need for more data on the clinical performance of other molecular assays such as the RT-Taqman PCR and the Nikisins qPCR. Immunoassays such as the antigen capture ELISA assays may be unsuitable reference standards due to the short period of antigenaemia during a Lassa virus infection. There is also evidence to suggest that the presence of IgM may either clear or mask the detection of antigens for Lassa in blood [20].

### Serum antigenaemia and antibody variation and the performance of Lassa diagnostic tests

Typically, serum antigen levels peak during the first week of illness with Lassa and are later replaced by IgM during the second week [20]. IgG levels start rising during the third week and remain positive even during the convalescent period [20]. In our included studies, the authors used samples collected from acutely ill patients, convalescent patients or did not clearly state the phase of infection during which samples were collected. It is very important for researchers that are evaluating the performance of serological assays to have this variation in serum antigens and antibodies in mind because the timing of testing can affect the performance of serological assays due to the variation in the serum antigen and antibody levels.

### The use of a point of care (POC) RDT for Lassa

The WHO call for rapid tests for Lassa has played a key role to incentivize diagnostic development for Lassa [15]. Even though a definitive diagnosis of Lassa is only possible in reference laboratories, point of care testing using RDTs has the advantage of reducing delays in testing and treating, as well as facilitating a better understanding of the nature of an outbreak [47]. Despite the urgent need for RDTs, our study identified only one RDT under evaluation: The Recombinant Lassa fever virus antigen test, which is a lateral flow immunoassay against the Josiah strain (lineage IV) of the Lassa fever virus. The performance of this assay was comparable to molecular tests such as qPCR and RT-PCR at temperatures 18–30 °C. These results are very promising given that at such level of performance, very few results will be false positives, making it very useful in improving the case definition during an outbreak [14]. Furthermore, having such a highly performant assay would greatly enhance appropriate patient enrollment for vaccine and therapeutic clinical studies [14].

Despite the high performance of the RDT, there are important concerns that may suggest the findings should be interpreted with caution. Firstly, there is not enough evidence to support the use of the Nikisins qPCR and Recombinant Antigen ELISA as the gold standards. Secondly, the performance of the test above 30 °C was not evaluated and given that Lassa fever occurs in humid areas with high temperatures (approaching 40 °C or more) such as in Northern Nigeria, these environmental conditions should be taken into consideration if the test is intended for use in such areas [5]. Thirdly, the assay was limited to the Josiah strain of the Lassa virus. Taking the diversity of the Lassa fever virus across West Africa into consideration, more work is needed to design and evaluate an RDT that can detect all Lassa strains. However, it has been reported that such a pan-Lassa virus assay is currently being evaluated in Nigeria [31]. Future efforts are also geared towards the development of a point of care multiplex molecular assay using the SHERLOCK (Specific High Enzymatic Reporter Unlocking) platform that can ensure even more precise testing with earlier detection as compared with the lateral flow immunoassay [48]. For such a point of care multiplex assay, more emphasis should be given to reducing the cost, to ensure affordability and high uptake by the governments of developing countries in West Africa.

### Antigen and antibody ELISA assays for detection of Lassa

For Serological assays to be useful in screening, etiological diagnosis, and sero-epidemiological studies of Lassa fever, they must be must be sensitive, specific and reliable, since a misdiagnosis can misguide public health interventions to control an outbreak and possibly trigger panic in the population [14]. The sensitivity and specificity of the single IgM ELISA assay evaluated were low using the same reference standard in two different studies conducted in Nigeria. Gabriel et al didn’t clearly state the phase of infection during which serum samples were collected and tested, while Ibekwe et al clearly included cases within 4 weeks of suspected infection. Even though Ibekwe et al were precise about their inclusion criteria, they did not stratify the number patients tested in each week after recruitment. Since IgM antibodies become detectable in the second week of infection [49], it is possible that the low performance of the IgM assay may have been due to the inclusion and testing of mainly patients in the first week of infection.

To improve on the performance of single antibody ELISA assays, Emmerich et al developed and evaluated the reverse ELISA techniques [24]. The sensitivity and specificity of the reverse IgG ELISA using Indirect Immunofluorescence assay (IFA) as gold standard were as high as RT-PCR assays. This high performance could be due to the modifications in the design of the assay that ensure a more direct binding of antigen-antibody complexes than in indirect ELISA assays [24]. The high performance of the reverse ELISA assays can be disputed based on the methodological flaws of the study. Using IFA as the gold standard, and the use of serum samples from healthy blood donors may suggest that the findings from the study are conservative and should be interpreted with care. Therefore, further studies evaluating the performance of the reverse IgG assay with serum from patients suspected of having Lassa at different stages in their clinical presentation are needed to either confirm or refute the findings.

The use of recombinant antigen detection of Lassa fever virus has improved on the development and access of Lassa fever diagnostics by obviating the need for BSL-4 facilities [28]. The Recombinant antigen ELISA tests showed a high sensitivity comparable with molecular testing. This higher performance of antigen tests compared to antibody tests has been linked to the high antigenemia in Lassa infection [50]. However, it should be noted that the short duration of antigenemia means antigens can become undetectable despite high levels of viremia in Lassa fever patients, thereby markedly reducing the performance of the antigen assays [14, 48]. Since antibodies have a longer duration in blood, one way that has been suggested to overcome this challenge has been to use combined antigen/antibody ELISA assays. This could explain why the combined antigen/IgM assay evaluated by Bausch et al had a higher performance than the single recombinant assay.

### For molecular assays

Several molecular assays have been developed for Lassa fever detection but only the performance of the GPC RT-PCR have been evaluated recently. The performance of the RT-PCR assay was very close to that of the virus culture used as gold standard. This can explain why RT-PCR assays have been used as clinical standard for in some Lassa fever studies [14]. The stark difference in sensitivity between conventional PCR and gene specific RT-PCR can be due to the high variability of the Lassa virus which limited the ability of the conventional PCR to reliably detect all strains. With the gene specific RT-PCR, a revised protocol which considered the 62 S RNA (ribonucleic acid) sequence from all Lassa endemic countries was used. This greatly improved the sensitivity of the RT-PCR. The main caveat of the study evaluating the performance of the gene specific RT-PCR is the small sample size used in the laboratory evaluation. Studies that are conducted in the field with larger sample sizes will be needed to confirm the findings. RT-PCR assays are widely used in screening samples and in reference laboratory settings in the diagnosis of Lassa, but the level of sophistication and training limit their use in peripheral health care settings. To overcome this limitation, a new RT-LAMP is currently under development, but a field evaluation is still yet to be carried out to provide data on its performance [27].

The main challenge with nucleic acid detection assays for Lassa is the genetic diversity which may have a profound negative effect on their performance even if there is minimal variations in just one primer [51]. However, by re-designing the assays after identification of mismatches, the performance of RT-PCR assays has been optimized. Furthermore, multiplex assays such as the RT-Taqman PCR and the PCR/LDR assays that detect several strains of Lassa alone or in combination with enzyme hybridization, as well as many other hemorrhagic viruses have been developed [29, 30]. Even though none of these multiplex assays have been evaluated in the field, their use in epidemic response will be crucial in screening patients in areas where many hemorrhagic fever viruses can occur and in which many patients may present with fever of unknown origin.

### Strengths and limitations

Our systematic review has several strengths and some limitations. Many databases were searched using a clearly defined search strategy. There was independent search, screening and extraction of data from articles, thereby reducing the bias in the selection of articles for inclusion. We also assessed the quality of included studies, through which weaknesses in the methodology of included studies were identified so that lessons can be learnt by other investigators as they design and conduct any future diagnostic studies for Lassa. The main limitation we could identify in our study was that we targeted only studies evaluating diagnostics for humans even though Lassa fever is a zoonotic infection [7]. Therefore, our study does not provide any information on the use of diagnostics for Lassa in animals. Despite this limitation, our study is the first of its kind involving the review of the evidence on a clearly formulated research question using systematic and explicit methods to identify, select and critically appraise relevant primary research, and to extract data from the studies on Lassa fever diagnostics. This high level of transparency and robustness suggests the level of bias is very low.

## Conclusion

Lassa fever has assays for antigenaemia, IgM, IgG and PCR detection. The RDT we identified in our study, reportedly performed well but more data are needed from other countries and at temperatures above 30 °C. Combined immunoassays perform better than the single IgM immunoassays. More data are needed to establish a clinical reference standard for Lassa fever diagnosis. Multiplex and pan-Lassa assays are needed for Lassa. More well conducted field and laboratory studies with a clear description of the patient/sample flow and timing are needed in future.

## Additional files

Additional file 1: **Table S1.** Medline search strategy. (DOCX 15 kb)

Additional file 2: **Table S2.** Embase search strategy. (DOCX 13 kb)

## Data Availability

All the data used in this study have provided in the tables submitted.

## Abbreviations

Ag: Antigen
ELISA: Enzyme Linked Immunosorbent Assay
IFA: Indirect Immunofluorescent Assay
LAMP: Loop-mediated Isothermal Amplification
qPCR: Quantitative PCR
QUADAS: Quality Assessment tool for Diagnostic Accuracy Studies
RDT: Rapid diagnostic test
ReELISA: Recombinant Enzyme Linked Immunosorbent Assay
RNA: Ribonucleic acid
RT-PCR: Real Time Polymerase Chain Reaction
